# Formation of pre-metastatic bone niche in prostate cancer and regulation of traditional chinese medicine

**DOI:** 10.3389/fphar.2022.897942

**Published:** 2022-08-17

**Authors:** Chiwei Chen, Renlun Huang, Jianfu Zhou, Lang Guo, Songtao Xiang

**Affiliations:** The Second Clinical College of Guangzhou University of Chinese Medicine, Guangdong Provincial Hospital of Chinese Medicine, Guangzhou, China

**Keywords:** pre-metastatic niche, bone metastasis, tumor microenvironment, prostate cancer, Traditional Chinese medicine

## Abstract

Prostate cancer with bone metastasis has a high cancer-specific mortality. Thus, it is essential to delineate the mechanism of bone metastasis. Pre-metastatic niche (PMN) is a concept in tumor metastasis, which is characterized by tumor-secreted factors, reprogramming of stromal cells, and immunosuppression by myeloid-derived suppressor cells (MDSC), which is induced by bone marrow-derived cells (BMDC) in the target organ. However, PMN does not explain the predilection of prostate cancer towards bone metastasis. In this review, we discuss the initiation of bone metastasis of prostate cancer from the perspective of PMN and tumor microenvironment in a step-wise manner. Furthermore, we present a new concept called pre-metastatic bone niche, featuring inherent BMDC, to interpret bone metastasis. Moreover, we illustrate the regulation of traditional Chinese medicine on PMN.

## 1 Introduction

Prostate cancer (PCa) has gradually become a major threat to humans. Recent reports reveal that 1.6 million people were diagnosed with PCa and 366 thousand people succumb to it each year (Pernar, Ebot, Wilson, & Mucci, 2018). PCa metastasis occurs predominantly in the bone, which is associated with high mortality (Mazzone et al., 2018; Riihimäki, Thomsen, Sundquist, Sundquist, & Hemminki, 2018), thereby suggesting the need for its mechanistic evaluation. Recent studies have focused on understanding the role of the pre-metastatic niche (PMN) and tumor micro-environment (TME) in cancer metastasis and have made a groundbreaking revelation about the mechanism of metastasis. However, little is known about specific bone metastasis, especially in PCa, because the PMN theory may not completely explain the metastatic tendency of PCa to the bone. Moreover, the bone TME seems more complicated than other secondary tumor TMEs. Therefore, we proposed a secondary concept of PMN: pre-metastatic bone niche (PMBN) to illustrate the mechanism of bone metastasis and bone TME. This review aims to explore the difference between PMN and PMBN and provide an insight into the formation of PMBN and the mechanism of bone metastasis in PCa.

## 2 Formation of the pre-metastatic niche

The classical hypothesis regarding metastasis is “seeds” (cancer cells) and the “soil” (host micro-environment) theory (Paget, 1989; Peinado et al., 2017). A hundred years later, this hypothesis has been further explained by the concept of PMN (Kaplan et al., 2005). With time, the theory of PMN has developed, where the formation of PMN takes place in three steps: first, migration of tumor-secreted extracellular vesicles and non-vesicular tumor-secreted factors to the future metastatic organ. Secondly, reprogramming of stromal cells occurs in the metastatic organ. Thirdly, recruitment of vascular endothelial growth factor receptor (VEGFR^+^) hematopoietic stem/progenitor cells (HSPC) that differentiate into myeloid-derived suppressor cells (MDSC) to exert immunosuppressive effects (Kaplan et al., 2005; Koh & Kang, 2012; Chin & Wang, 2016; Zheng et al., 2020). PMN can provide a hotbed for metastatic cells. However, different tumors display varying propensities to target organs. According to a recent report, 50% of breast cancer, 44–90% of pancreatic cancer, and 35–55% of colorectal cancer are known to migrate to the liver. About 30–60% of breast cancer migrated to bone, and surprisingly, 68–80% of PCa migrated to bone (Zhuyan et al., 2020). Till date, the mechanisms of specific metastasis are poorly understood. As the tumor micro-environment and the immune environment have been explored, the mechanism of specific metastasis could be explained in this aspect, especially the bone metastasis of PCa.

### 2.1 Pre-metastatic niche vs. pre-metastatic bone niche

PMN has been detected in the lung and liver, but is poorly explored in bone. However, the components of PMN like bone marrow-derived cells (BMDC)/HSPC are components of the stromal niche and hence do not require reprograming like other PMN. Thus, we put forth a concept of PMBN to explain the specific metastasis, particularly in PCa. We discuss the differences in the sources and functions of components between PMN and PMBN (Table 1).

**TABLE 1:** The difference of source and function of components between PMN and PMBN.

Components	PMN	PMBN	Ref.
BMDC/HSPC	Migrate from bone	Inherent	Kaplan et al. (2005); Kaplan, Psaila et al. (2006)
CXCL12	Secreted by stromal cells, fibroblasts and epithelial cells	Secreted by stromal cells, fibroblasts, epithelial cells, osteoblast, osteoclast	Ahmadzadeh et al. (2015); Meng, Xue, & Chen (2018)
extracellular matrix	Collagens, proteoglycans, laminins, fibronectin, matricellular-associated proteins	Collagens, proteoglycans, laminins, fibronectin, matricellular-associated proteins, osteopontin	Mouw et al. (2014); Gartland et al. (2016); Kai et al. (2019)
bone morphogenetic protein (BMP)	Inhibits breast cancer cells at lung metastatic sites	Promote invasive properties in prostate cancer at bone metastatic sites	Graham et al. (2010); Gao et al. (2012)
Parathyroid hormone-related protein	None	Upregulating the expression of integrin	Shen & Falzon (2003)
bone-stored growth factors	None	Includes insulin-like growth factors, TGF-β	Yoneda (2011); Xie, Ling, van Dam, Zhou, & Zhang (2018); Hiraga (2019)

### 2.2 The two subtypes of myeloid-derived suppressor cells

MDSCs are a heterogeneous population of immature myeloid cells (IMCs), which are the precursors of dendritic cells (DCs), macrophages, and granulocytes. They have the ability to significantly inhibit immune cell responses and negatively regulate immunity. MDSCs are mainly composed of two subtypes, including polymorphonuclear myeloid-derived suppressor cells (PMN-MDSCs) and monocytic myeloid-derived suppressor cells (M-MDSCs) (Li et al., 2021). Mice and human MDSCs have different cell surface markers (Gabrilovich, 2017). In mice, PMN-MDSCs and M-MDSCs can be respectively defined as CD11b^+^Ly6G^+^Ly6C^low^ and CD11b^+^Ly6G^−^Ly6C^hi^ (Gabrilovich, 2017). Similarly, PMN-MDSCs and M-MDSCs can be respectively defined as CD11b^+^CD14^−^CD15^+^/CD11b^+^CD14^−^CD66b^+^ and CD11b^+^CD14^+^HLA^−^DR^-/low^CD15^−^ in human peripheral blood mononuclear cells (PBMC) (Gabrilovich, 2017). Increasing evidence shows that M-MDSCs and PMN-MDSCs achieve immunosuppressive effects through different mechanisms. PMN-MDSCs highly express arginine (Arg1) and reactive oxygen species (ROS), while M-MDSCs highly express nitric oxide (NO) and nitric oxide synthase (iNOS), all of which mainly inhibit the function of T cells (Gabrilovich, 2017). Notably, current research suggests that PMN-MDSCs are the main subtype responsible for promoting prostate cancer metastasis. For example, one study has found that PMN-MDSCs are more abundant in bone metastases than in primary prostate cancer (Wen et al., 2020).

### 2.3 Steps for the formation of the pre-metastatic bone niche

#### 2.3.1 Step 1: Tumor-derived soluble factors and tumor-derived exosomes are induced by hypoxia and inflammation

TDSF such as interleukin (IL)-10, vascular endothelial growth factor (VEGF), transforming growth factor-beta (TGF-β), and soluble human leukocyte antigens (HLA) molecules have several important immune modulatory functions like immune escape, immunosurveillance, and subduing the immune cells’ functions (Allard et al., 2011; Deepak & Acharya, 2010; Packard, Lee, Remold-O'Donnell, & Komoriya, 1995; Shimabukuro-Vornhagen et al., 2012). As the first step of the formation of PMBN, the role in bone environment and MDSC has been reported. Prostate-derived soluble factors block osteoblast differentiation (Martínez, Silva, & Santibáñez, 1996), Intriguingly, TDSFs promote the process of differentiation of bone marrow-derived mesenchymal stem cells into mature osteoblasts cultured using mineral-containing 3D poly scaffolds (Lynch et al., 2016). TDSFs play an important role in the differentiation of CD11b-Gr1-bone marrow progenitor cells into MDSCs (Morales, Kmieciak, Knutson, Bear, & Manjili, 2010; Ishii et al., 2018). Notably, the recruitment of HSPC and their differentiation into MDSC are symbolic events responsible for PMN formation (Zheng et al., 2020) as above. Furthermore, TDSF can recruit the regulatory T cells (Tregs) (Du & Wang, 2011; Crane, Ahn, Han, & Parsa, 2012) that account for the suppression of immune cells and form an immunosuppressive microenvironment, which may protect tumor cells from immunotoxicity. This evidence provided strong evidence about the regulation of TDSF in the formation of PMBN. Communication *via* exosomes between primary cancer cells and the microenvironment of target organs is vital for PMN formation and metastasis (Wortzel, Dror, Kenific, & Lyden, 2019), especially tumor-derive exosomes (TDEs) that carry out functions such as organotrophic metastasis, restraining cancer immune surveillance, removing metabolic waste, remodeling distant PMN, and promoting tumor invasion (Chalmin et al., 2010; Deep et al., 2020; King, Michael, & Gleadle, 2012; Y. Liu & Cao, 2016; Panigrahi et al., 2018). Essential functions are associated with these proteins expressed by the TDEs at their surface. For example, heat shock protein 72 expressed by TDEs suppresses cancer immune surveillance by enhancing MDSC suppression *via* transducers and activators of transcription (STAT) signaling pathway (Chalmin et al., 2010). Thus, TDEs and PMNs have a synergistic effect on immunosuppression by promoting MDSC function. Moreover, an experimental study that unveiled the mechanism of organotrophic metastasis by TDEs examined the lung-tropic, liver-tropic, and brain-tropic exosomes by quantitative mass spectrometry. They found that integrins, an important component in PMN, representing the cell adhesion receptor proteins in exosomes, were the deciding factor for metastatic organotropism. They showed that ITGα6β4 and ITGα6β1 expressed by lung-tropic exosomes migrated to the lung microenvironment and that pancreatic-tropic exosomes expressing ITGαvβ5 preferred liver niches (Hoshino et al., 2015; Y. Liu & Cao, 2016). However, integrins expressed by TDEs migrating to the bone still need to be explored.

Hypoxia and inflammation driven by PCa are the two major causes for the secretion of TDSF and tumor-derived exosomes. Tumor promotes progression and metastasis potentially through exosome driven by hypoxia which may be mediated by hypoxia inducible factor-1α (King et al., 2012). Meanwhile, the data in PCa cells was more representative. Recent data showed that PCa derived exosomes promoted invasiveness and stemness under hypoxia, which in turn promoted the activity of matrix metalloproteinases (MMPs) (Deep et al., 2020). Interestingly, the expression of MMP is one of the features of PMN; thus, the hypoxia-tumor derived exosomes axis plausibly remodels distant PMN (Deep et al., 2020). Moreover, a large number of exosomes are secreted by PCa cells, which is a mechanism to remove metabolic waste and promote the survival of PCa under chronic hypoxia (Panigrahi et al., 2018). Other molecules induced by hypoxia, including lysyl oxidase, annexin A1, and PIM kinases (Erler et al., 2009; Bizzarro et al., 2017; Toth et al., 2019), also regulate the invasion of PMN. Inflammation is not only induced but is also a part of the TME, which is rich in proinflammatory cytokines and cells. These promote metastasis in several ways, such as inducing TDSF, which can potentially alter myelopoiesis (Ugel, De Sanctis, Mandruzzato, & Bronte, 2015). In PCa cells, TDSF such as indoleamine 2,3-dioxygenase and IL-6 have already been reported to mediate PCa morbidity. Elicitation of PCa-TDSFs by inflammatory factors like interferon gamma (IFN-γ) and TNF-α might be responsible for rendering a tumor untreatable (Banzola et al., 2018). Another TDSF called sHLA-E can be upregulated by IFN-γ, TNF-α and IFN-α (Allard et al., 2011). Further, IL-6 secretion regulates the epithelial-mesenchymal transition (EMT) and homing of tumor cells to the bone (Nguyen, Li, & Tewari, 2014).

#### 2.3.2 Step 2: Migration of metastasis-initiating cells

MICs, stimulated by TDSF and TDEs, are recruited to the bone metastatic lesions, preparing to form the PMBN. They display long-term self-renewal, specific driver mutations, and high cellular plasticity (Celià-Terrassa & Kang, 2016). Although tumor metastasis is prevalent in clinics, dissemination of the tumor to a secondary lesion at a molecular level is difficult. Metastasis is a difficult process with a huge rate of attrition; thus, only about 1% of disseminated tumor cells (DTCs) are estimated to metastasize successfully (Luzzi et al., 1998; Cameron et al., 2000). MICs are somewhat like cancer stem cells that maintain their stemness and self-renewal. However, their most prominent feature is dormancy. The dormant MIC travels to distant metastatic target organs, like bone, becomes a part of PMN or PMBN, and when the PMN/PMBN matures, the MIC resuscitates and exerts its malignant potential. However, MIC in dormancy still plays a critical role, especially in bone. Bone marrow contains hematopoietic niches that also play an important part in bone metastasis, especially in the dormant stage of MIC. Mesenchymal and endothelial cells contribute to a microenvironment called hematopoietic niches (Aurrand-Lions & Mancini, 2018), which is a protective site for tumor cells. Cancer cells survive in a dormant state by utilizing hematopoietic niches (Mukaida, Zhang, & Sasaki, 2020). PMN is suggested to be created or conditioned by early DTC which might affect metastasis development even through dormancy (Sosa, Bragado, & Aguirre-Ghiso, 2014). When MICs migrate to the bone marrow, osteogenic cells form heterotypic adherens junction with MICs and promote outgrowth. MICs also use secreted and membrane-bound vascular cell adhesion molecule 1 (VCAM1) to recruit pre-osteoclasts, which can further promote osteolytic invasion of indolent bone micro-metastasis by connecting α4β1-positive osteoclast progenitors (Lu et al., 2011; Celià-Terrassa & Kang, 2016). Besides, PCa-induced osteoblast activity increases receptor activator of nuclear factor kappa-Β ligand (RANKL), release of parathyroid hormone, and promotes osteoclast activity (Casimiro, Ferreira, Mansinho, Alho, & Costa, 2016). The above findings demonstrate that PMBN MICs enhance the “vicious cycle” of bone metastasis, which is another difference between PMBN and PMN.

#### 2.3.3 Step 3: Activation of chemokine-chemokine receptor signaling

##### 2.3.3.1 Chemokine (C-X-C motif) ligand 12/receptor 4 axis in bone

Stromal derived factor (SDF)-1, also called CXCL12, is highly expressed within the fully formed PMN (Kaplan et al., 2006a) and whose secretion is an important event for PMN. Here, we discuss some reports on CXCL12/CXCR4 in bone metastasis of PCa. CXCL12, secreted by osteoblast, endothelial cells, and mesenchymal stem cells, plays a vital role in the formation of the bone metastatic niche (Ahmadzadeh et al., 2015). CXCL12 is known to be more highly expressed in metastatic lesions than in normal tissues of PCa (Sun et al., 2003). Chemokines recruit cells with corresponding receptors by generating a concentration gradient (Jin, Xu, & Hereld, 2008). PCa were found to express CXCR4 (Sun et al., 2003), and the CXCL12 secreted by osteoblast and osteoclast then recruits the PCa cells to the PMBN. This may be a possible mechanism for cancer cells’ “homing” to the bone and may be responsible for their growth in selected organs (Sun et al., 2003). CXCL12 levels were found to be high in the pelvis, tibia, femur, liver, and adrenal/kidneys compared with those in the lungs, tongue, and eye (Sun et al., 2005). Interestingly, tumor-derived CXCL8 and phosphatase and tensin homolog (PTEN)-deficient cancer cells increase their sensitivity and reactivity to stromal chemokines by upregulating the expression of receptors in cancer cells and inducing stromal chemokine synthesis (Maxwell, Neisen, Messenger, & Waugh, 2014). PTEN is a regulator of CXCL12/CXCR4 and its loss leads to the activation of both Akt1 signaling and MMP9 expression. This promotes the expression of CXCL12/CXCR4, which in turn regulates the metastasis and invasion of PCa. Moreover, Akt1 overexpression reversed the osteosclerotic phenotype to an osteolytic phenotype and promoted intra-osseous tumor growth (Chinni et al., 2006; Conley-LaComb et al., 2013).

##### 2.3.3.2 Tumor-associated macrophages/chemokine (C-C motif) ligand 5 and tumor-associated macrophages/CXCL1 axis in bone

Chemokines make a great contribution to the PMN/PMBN. Here, we discuss two important axes in the bone micro-environment. TAMs are immune cells that have anti-tumor properties. However, the M2 phenotype of TAMs reveals a contrasting function, promoting tumor angiogenesis and metastasis (S. Lin et al., 2017). A study shows that consumption of M2 TAMs disrupted lung PMN and prevented metastasis (Chen et al., 2017b). TAMs function by secreting chemokines such as CCL5 and CXCL1. A recent study showed that TAMs/CXCL1 signaling could enhance breast cancer metastasis (N. Wang et al., 2018) and stimulate the recruitment of HSPCs and their differentiation into MDSCs, further promoting the formation of PMN (S. Wang et al., 2020; Zheng et al., 2020). However, TAMs/CXCL1 has not been reported in bone metastasis. Surprisingly, CCL5 was found to be a critical chemokine in gastric cancer, colorectal cancer, and breast cancer (An et al., 2019; Ding et al., 2016; S. Zhang et al., 2018). TGF-β signaling is an important pathway regulating the bone micro-environment and crosstalks with several pathways associated with tumor invasion (we will discuss the TGF-β later). CCR5, the cognate receptor of CCL5, was shown to be regulated by TGF-β signaling (S. Lin et al., 2017). Also, THP1-derived TAMs co-injection with cancer cells increased the bone metastasis of PCa xenografts, while CCL5 knockdown partly abrogated it (R. Huang et al., 2020).

##### 2.3.3.3 Angiopoietin-1/tie-2 axis in bone

Ang-1 and its receptor Tie-2 are one of the significant cytoactive molecules found within bone marrow, primary lesion and the PMN (Kaplan et al., 2006b). Tie-2 expression was found to be higher in PCa cell lines, which are capable of migrating to the bone. The Tie-2 high PCa cells displayed more adherence than the Tie-2 low PCa cell population to both osteoblasts and endothelial cells, and these cells also had a high expression of cancer stem cells (CSCs). However, knockdown of the Ang-1 led to suppression of CSCs. More importantly, only the Tie-2 high but not the Tie-2 low cells developed metastasis *in vivo* (K. D. Tang et al., 2016). Moreover, ng-1 mRNA expression was not observed in bone, lymph node or liver metastasis but was observed in bone marrow cells (Morrissey et al., 2008), suggesting that PCa cells move to PMBN through the Ang-1/Tie-2 axis.

#### 2.3.4 Step 4: Changes in extracellular matrix for preparing pre-metastatic bone niche

ECM changes are symbolic pre-metastatic changes in the target organ (Zhuyan et al., 2020) and contain comprehensive components such as collagens, proteoglycans, laminins, fibronectin, and matricellular-associated proteins (Mouw, Ou, & Weaver, 2014; Kai, Drain, & Weaver, 2019). When it comes to PMN, hypoxia, chemokines, and TDSF, as discussed previously, induce ECM to change to a form with increased stiffness and tensile strength (Gartland, Erler, & Cox, 2016). Furthermore, lysyl oxidase-mediated collagen crosslinking creates a fibrotic microenvironment supporting metastatic growth (Cox et al., 2013). Notably, each organ has its own PMN and changes in ECM, but when it comes to PMN and bone, the whole organ (bone) is an enormous “organized mesh” containing a tremendous amount of ECM, which consists of type I collagen, fibrous proteins, and non-collagenous proteins predominantly (Gartland et al., 2016). This is another characteristic of PMBN which is different from PMN, giving an insight into why so many tumors have a predilection for bone metastasis. An enzyme called lysyl oxidase is known to post-translationally modify collagen and elastin in the ECM, thereby catalyzing the covalent crosslinking of collagen fibers associated with bone metastasis (Gartland et al., 2016). An *in vivo* experiment showed that tumor bearing mice displayed increased bone loss and formed focal osteolytic lesions over time before metastasizing, and these changes were lysyl oxidase (LOX)-dependent (Gartland et al., 2016). Thus, during PMBN formation, bone is osteolytic, far before the time that cancer cells home to the bone. This is yet another feature of PMBN. Another important ECM component in the bone is osteopontin, which is associated with malignant transformation and acts as a paracrine and autocrine mediator of PCa growth and progression (Thalmann et al., 1999). As stated previously, the bone marrow microenvironment contains intricate components including ECM, cytokines and chemokines regulate the hematopoietic progenitor cells’ proliferation and differentiation, which was a core step of PMBN.

#### 2.3.5 Step 5: Recruitment of VEGFR1^+^ bone marrow-derived cells in bone

Firstly, VEGFR was found to have a high expression in PCa. More importantly, its expression was elevated at sites of bone metastasis compared to the original prostate tumor. VEGF interacting with VEGFR regulated adhesive and migratory properties of the cancer cells (J. Chen, De, Brainard, & Byzova, 2004). As discussed about PMN formation above, HSPC expressing VEGFR1 and bone BMDC colonize pre-metastatic sites before tumor cells (Kaplan et al., 2005). However, the reason for the recruitment of VEGFR+ BMDC or VEGFR+ HSPC to be considered as a symbolic event of PMN is not known (Kaplan, Rafii, & Lyden, 2006; Zheng et al., 2020)? The following reasons can be considered: regular T cells and MDSC, especially those differentiated from HSPC, are functional types of BMDC (Koh & Kang, 2012). Purified HSPC *in vivo* differentiated into MDSC in early metastatic sites of tumor-bearing mice and promoted tumors metastasis (Giles et al., 2016). The MDSC in the PMN provided a microenvironment suitable for cancer cells by enhancing immunosuppression, leaking vasculature, and collagen restructuring in the PMN by suppressing T cells, generating a lot of NO, arginase1 (Arg-1) and immunosuppressive cytokines, and promoting regular T cell expansion (Y. Wang, Ding, Guo, & Wang, 2019). A recent study reported that, during tumor progression, MDSC contributed to PMN formation by upregulating MMP-9 expression (J. Zhang et al., 2020). The ability to suppress immune cells is an important standard that is used to define MDSC (Bronte et al., 2016). Recruitment of HSPC as a symbolic event for PMN, which has been proven by several cancers like breast and colorectal cancer (Psaila, Kaplan, Port, & Lyden, 2006; C. Zhang et al., 2014). As for bone metastasis, distant primary tumor drives the expansion of HSPC within the bone marrow and their mobilization to the bloodstream (Giles et al., 2016). That means they don’t even need to move to the bloodstream and site to the PMN, because the bone is already its PMN, we call that PMBN. The bone marrow is an inherent store of HSPC. Besides, metabolic conditions in niches such as calcium concentrations mediated HSPC retention within, but not homing to, the endosteal niche (Kucia et al., 2005). It is not surprising that many cancers show a proclivity to establish themselves in the bone marrow (Kaplan, Psaila, et al., 2006). Recently, a new study demonstrated a previously unidentified role for perivascular cells in PMN formation, which is a new mechanism for PMN. The author showed that perivascular cells lost the expression of traditional vascular smooth muscle cells to build the PMN by stimulating the tumor-secreted factors and genetic activation of Klf4 (Murgai et al., 2017).

#### 2.3.6 Step 6: Integrin plays a role in the last step of the pre-metastatic bone niche

**FIGURE 1:**
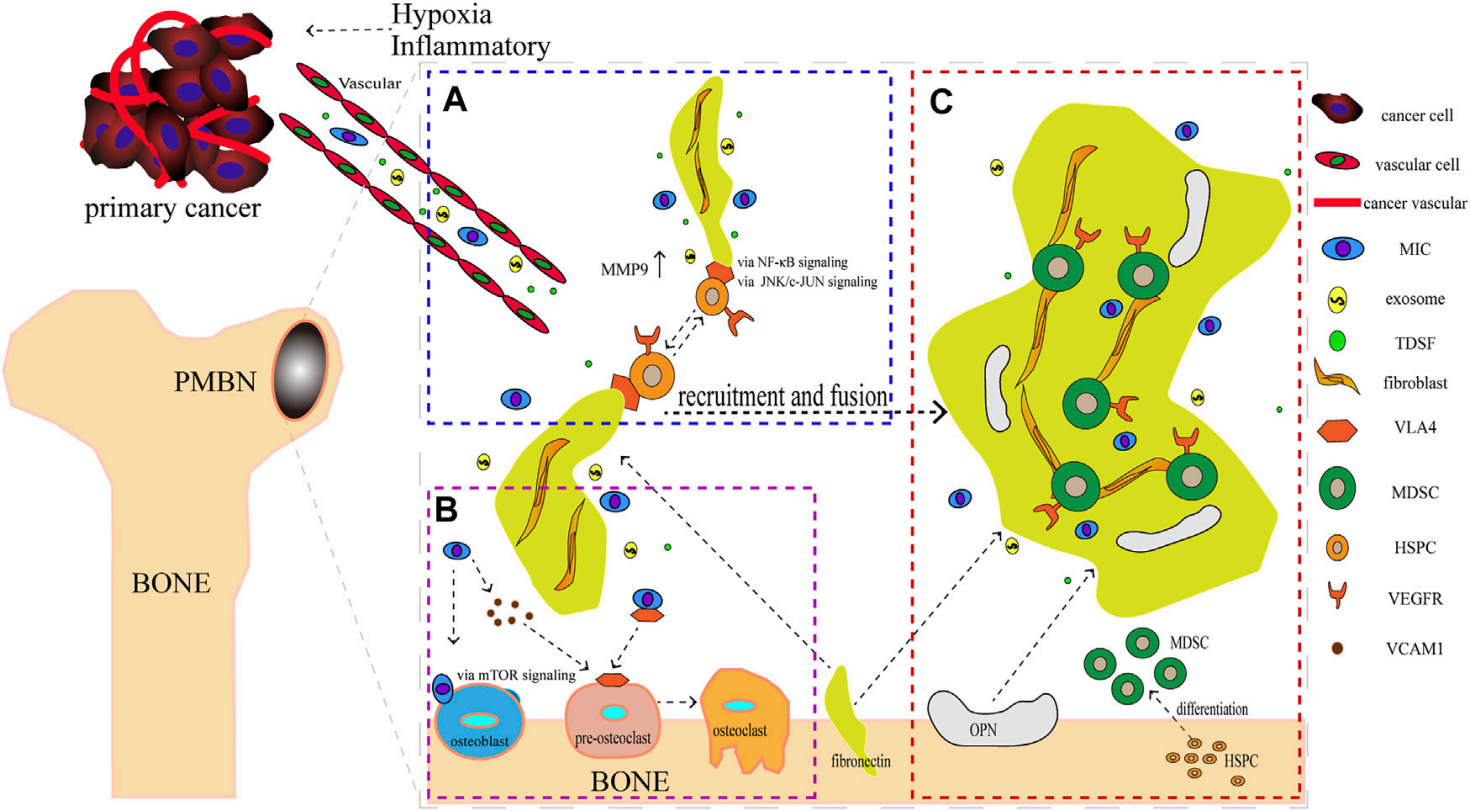
The formation of the Pre-Metastatic Bone Niche (PMBN). **(A)** Metastasis-initiating cells (MICs), exosomes, and tumor-derived soluble factors (TDSF) were secreted by cancer cells under hypoxia and inflammatory conditions. Fibronectin carried by VEGFR + HSPC, which was connected by integrin alpha4 beta1 (VLA4), were recruited together. **(B)** Before bone metastasis, the balance of osteoblast and osteoclast is disrupted. MICs can adhere to osteoblasts and recruit pre-osteoclasts *via* vascular cell adhesion protein 1 mediated by mTOR signaling. This event promotes the formation of osteoclasts. **(C)** Maturation of PMBN. Notably, HSPC was differentiated into MDSC. The difference of source and function of components differ between PMN and PMBN.

Here, we illustrate some data about integrin in the bone environment of PCa. Bone-metastatic castration-resistant PCa is lethal and shows drug resistance. One of the mechanisms is the integrin α6β1-mediated adhesion to laminin (Toth et al., 2019). Adhesion capability of PCa cells to bone marrow endothelial cells was enhanced through upregulation of integrin-α4 expression, concurrent with transcriptionally activated NF-κB (Zhao et al., 2020). Parathyroid hormone-related protein, upregulates the expression of alpha1, alpha5, alpha6, and beta4 integrin subunits and plays a vital role in the development of bone metastasis (Shen & Falzon, 2003). In other cell lines, integrin β1 has high expression in hepatocellular carcinoma with stiffness substrates and co-regulates with the JNK/c-JUN signaling pathway in upregulating LOXL2, MMP9, fibronectin production, CXCL12 expression and BMDC recruitment, which account for PMN formation (Wu et al., 2018). However, the positive outcome of alpha4 integrin which is most relative with PMN. It has been reported that fibroblasts contributed to breast cancer bone metastasis by mediating CCL4/CCR5 axis (Sasaki et al., 2016). Upregulation of fibronectin activated by fibroblasts is one of the earliest changes observed in future metastatic niches (Kaplan et al., 2005), and then integrin alpha4beta1 (VLA4) carrying VEGFR1^+^ HSPC attached to the upregulating fibronectin. From the figure of Kaplan, VLA4 connected with HSPC to fibroblasts and that was the last step of the PMN formation (Kaplan, Rafii, et al., 2006). Those series events associated with VLA4 are similar to their function within the bone marrow (Kaplan, Psaila, et al., 2006). It seems like the PMN try to provide a tumor-friendly environment by imitation from bone marrow environment and PMBN is an integration of bone marrow environment. Consequently, we know from above that PMBN is the initial PMN. In PCa cells line, VLA4 is not the only key for connection, integrin α6β1, integrin alpha1, alpha5, alpha6, and beta4 had their contribution to the bone metastasis. VLA4+VEGFR + HSPC attaching fibronectin completely is a symbolic event of the maturation of PMBN (Figure 1).

### 2.4 From pre-metastatic bone niche to mature bone metastasis

#### 2.4.1 Activation of migration of Metastasis-initiating cells

Reactive oxygen-generating enzyme (Nox1) increases tumorigenicity of prostate epithelial cell line. Importantly, Nox1 could significantly upregulate VEGF mRNA, induce VEGFR1, VEGFR2 and MMPs (Arbiser et al., 2002), which is important for PMN and MIC. Nox1 may activate MIC by triggering the angiogenic switch. Early DTCs contributed to the PMN and the later ones account for the escape from dormancy to promote metastasis (Sosa et al., 2014). For example, tumor cells could escape from dormancy by upregulating and activating VCAM1 (Lu et al., 2011). Periostin, as a cancer-promoting factor, is induced by endothelial tip cells, also promoting dormancy escape (Malanchi et al., 2011; Ghajar et al., 2013). β1 integrins are indispensable to prevent dormancy onset and critical for proliferation of micro-metastatic cancer cells (Shibue & Weinberg, 2009; Sosa et al., 2014). Gaining the function of EMT and MET is also a typical event of MICs’ activation. MICs are a type of stem-like cell that increases expression of EMT, stemness of stem cells and pro-survival (Lawson et al., 2015). When they arrive at the secondary organ, a reversed process: MET is required (Tsai, Donaher, Murphy, Chau, & Yang, 2012). Recent research found that low-burden cells expressed high-level dormancy genes such as CDKN1B, CHEK1, TGFBR3 and TGFB2 while higher-burden metastatic cells rarely expressed dormancy-associated genes, whereas highly expressed cell-cycle promoting genes such as CDK2, MYC, MMP1, and CD24, which were accounted for dormancy escape (Lawson et al., 2015). We could imply that during the formation of PMBN, the surrounding MIC belong to low-burden cells which show the stemness however, when the PMBN is completely finish, those MIC turn to more heterogeneous. Energy metabolism is another aspect to elucidate the activation of MIC. A study demonstrated that in brain metastatic breast cancer cells, tumors could obtain energy in multiple ways in order to reinitiate MIC proliferation (Celià-Terrassa & Kang, 2016; E. I. Chen et al., 2007). A high plasticity in energy substrate metabolism in PCa maybe another energy mentalism pathway to active MIC as previous reported (Aguilar et al., 2016). Other mechanism such as high expression of VCAM1 induced by inflammatory, inducing reactivation of growth by stromal niche also account for the activation of MIC (Lu et al., 2011; Giancotti, 2013). Intriguingly, bone morphogenetic protein (BMP) could promote invasive properties in PCa at bone metastatic sites (Graham, Agrawal, & Abdel-Mageed, 2010), but the BMP inhibitor Coco reactivates breast cancer cells at lung metastasis. It seems that BMP plays an opposite role in PMBN and PMN (Gao et al., 2012).

#### 2.4.2 Disruption of balance between osteoblast and osteoclast

The balance comes from a classical theory of bone metastasis called the “vicious cycle theory,” which demonstrates a series of molecular events. In brief, osteoclast-stimulating factors, such as PTHrP, promote osteoblasts to secrete RANKL, which in turn promotes differentiation of osteoclast precursor cells into mature osteoclasts (Mundy, 2002; Hiraga, 2019). Later, osteoclasts secrete TGF-β to promote PCa invasion and migration. Surprisingly, a lot of insights go into exploring PCa with bone metastasis. First comes the regulation of micro-RNA. Three studies showed that micro-RNA (miR-141-3p, miR-210-3p, miR-133a-3p, miR-204-5p) affected bone metastasis by activating NF-κB and PI3K/AKT signaling (S. Huang et al., 2017; Ren et al., 2017; Y. Tang et al., 2018; Wa et al., 2019; Ye et al., 2017). Micro-RNAs (microRNA-124, miR-133b, miR-505-3p, miR-19a-3p) regulate the bone metastasis *via* activating TGF-β signaling pathway (Coniglio, 2018; S. Huang et al., 2018; Y. Tang et al., 2019; Wa et al., 2018). Other factors such as lnc-RNA, IFITM3, PICK1were also mediate TGF-β signaling pathway (Y. Dai et al., 2017; Lang et al., 2020; X. Liu et al., 2019). TGF-β signaling is an important signaling/factor for bone metastasis in PCa. To echo the PMBN, those symbolic factors of PMBN indeed play a role in bone metastasis. For example, VEGF contributes to PCa-induced osteoblastic activity *in vivo* (Kitagawa et al., 2005); MMP2 is upregulated to promote PCa bone metastasis (Dutta et al., 2014; Chen et al., 2017a). Other factors, like interferon regulatory factor 7, stimulate oncostatin M or even (m6A) mRNA methylation, regulate the bone metastasis of PCa. Here, we highly focus on the specific factors of bone: bone-stored growth factors. Osteoclastic bone resorption followed by the release of bone-stored growth factors (Yoneda, Hashimoto, & Hiraga, 2003; Yoneda, 2011) such as insulin-like growth factors and TGF-β therefore, provides fertile soil for metastatic cancer cells. Bone-derived IGF-I connected with bone and metastasized the tumor cells *via* IGF-IR/Akt/NF-κB signaling, while BMP9 was able to inhibit the migration involving SDF-1/CXCR4-PI3K pathway, which was associated with PMBN regulation (Hiraga et al., 2012; W. Wang et al., 2015).

#### 2.4.3 Oteoblastic bone metastasis in pre-metastatic bone niche

Oteoprotegerin (OPG)/RANKL/RANK axis mediated osteolytic bone metastasis is common in a lot of cancer bone metastasis, but there is a consensus that osteoblastic bone metastasis is dominant in PCa (Berruti et al., 2001). OPG is an important cytokine to prevent pre-osteoclast from becoming osteoclast by acting as a decoy receptor for RANKL. A meta-analysis found that OPG was highly expressed in PCa with bone metastasis (Zang et al., 2015), suggesting its importance in mediating osteoblastic bone metastasis. Dickkopf-1 (DKK-1)/Wnt signaling, endothelin-1 (ET-1) and BMP are also very important in regulating osteoblastic bone metastasis. In TME, PCa cells highly express DKK-1 to promote cancer proliferation before bone metastasis. However, when the PMBN is accomplished, PCA cells rarely express DKK-1 (Aufderklamm et al., 2018), which subsequently activates Wnts’ osteoblastic activity (Schwaninger et al., 2007; Hall, Daignault, Shah, Pienta, & Keller, 2008). Moreover, endothelin 1, elicits pleiotropic effects on the microenvironment, expressed by PCA cells, suppresses the function of DKK-1 so as to enhance the activation of Wnt signaling (Rosanò, Spinella, & Bagnato, 2013). BMP, which induces MIC to activate cancer cells in PMN to mature PMBN, also plays a role in promoting osteoblastic activity (J. Dai et al., 2005). Osteoblast enhanced VCAM-1 expression in PCa cells and subsequently promoted the adherence of cancer cells to osteoblasts (Chang et al., 2018) and surprisingly, this process was corroborated with the formation of PMBN.

### 2.5 Traditional Chinese medicine and Pre-metastatic niche

The theoretical basis of treatment using Traditional Chinese Medicine (TCM) is the Yin-Yang theory. It is important for TCM to maintain the Yin-Yang balance in the body. Tumor cells are taken for Yang as invasion and spread, while immune cells are taken for Yin on account of clearing. If immune cells cannot clear tumor cells, it will cause an imbalance of Yin-Yang in the body, leading to disease progression. Notably, MDSCs are the main cells that make up PMBN and are capable of forming a pre-metastatic immunosuppressive microenvironment (Zheng et al., 2020). Fortunately, TCM with multi-targeted efficacy can exert anti-tumor effects by regulating MDSCs. For example, cinnamaldehyde (CA), an important component of cinnamon, can enhance the immune killing effect of PCa by inhibiting myeloid-derived suppressor cells (MDSCs) (Han et al., 2020). Accumulated evidence indicates that TCM plays a pivotal role in regulating the pre-metastatic niche and suppressing tumor metastasis. Thus, PMN is an integral process involving multiple organs, multiple cells, and multiple cytokines. Coincidentally, TCM has a holistic view of diagnosis and treatment. In the past few years, a large number of studies have shown that TCM can suppress tumor metastasis by inhibiting the formation of PMN in breast cancer (Tian et al., 2020; Zheng et al., 2020), gastric cancer (Zhu, Zhou, Xu, & Wu, 2017) and colorectal cancer (C. Chen et al., 2019). Therefore, summarizing the functions of TCM herbs that can inhibit the formation of PCa PMN is beneficial to extend the clinical application of TCM.

Several studies have demonstrated that natural phytochemicals extracted from TCM herbs show great advantages in the control of tumor metastasis *via* inhibiting the formation of PMN. Active components extracted from TCM herbs such as bufalin, which were obtained from the ChanSu skin and parotid venom glands, can inhibit bone metastasis of PCa (J. J. Zhang et al., 2019). Celastrol, one of the active components of Tripterygium wilfordii, can inhibit the bone metastasis of PCa cells by inhibiting the VEGF pathway of bone marrow-derived endothelial progenitor cells (BM-EPCs) (Kuchta et al., 2017). However, these two studies only preliminary explored the inhibition of bone metastasis of TCM herbs, and have not thoroughly investigated the mechanism of inhibiting bone metastasis of PCa. The balance of osteogenesis and osteoclast is an important regulatory mechanism for the formation of PMN in bone metastasis (Furesi, Rauner, & Hofbauer, 2021), and the RANKL pathway is closely related to osteogenesis (Portal-Núñez et al., 2017). Perilla aldehyde (PAH), one of the active components of the TCM herb Perilla, is widely used and has important anticancer activity. A study has found that PAH can inhibit the formation of an osteoclast pre-metastatic niche by inhibiting the RANKL pathway and ultimately inhibit the bone metastasis of PCa (Z. Lin et al., 2022). In addition, Aldehydic components of Cinnamon bark ultimately extract can also suppress RANKL-induced osteoclastogenesis by down-regulating the expression of transcription factor NFATc1 (Tsuji-Naito, 2008).

However, there is still a lack of in-depth research on the regulation of PCa bone pre-metastatic niche by TCM. In addition to osteoclastogenesis, some immunosuppressive cells such as MDSC and Treg play an important role in the formation of the PCa bone pre-metastatic niche (Cheng & Wang, 2021). There are some exciting progresses in the field of breast cancer pre-metastatic niche. For example, Wang et al. found that XIAOPI formula, a TCM herb composed of multiple prescriptions, can inhibit the pre-metastatic niche formation in breast cancer *via* the suppressing function of TAMs (Zheng et al., 2020). TCM treasure trove. On the basis of illuminating the formation of the PCa bone metastasis niche, in-depth research on the mechanism of TCM will help us to expand the clinical application of TCM in the treatment of patients with PCa bone metastasis (Table 2).

**TABLE 2:** Chemical structure and function of traditional Chinese medicine monomer in PMN.

Compound	Structure	Function	References
Bufalin	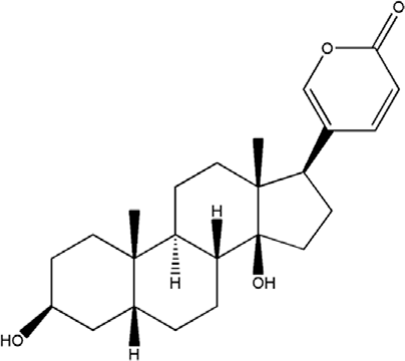	1. Inhibit bone metastasis of prostate cancer	J. J. Zhang et al. (2019)
Celastrol	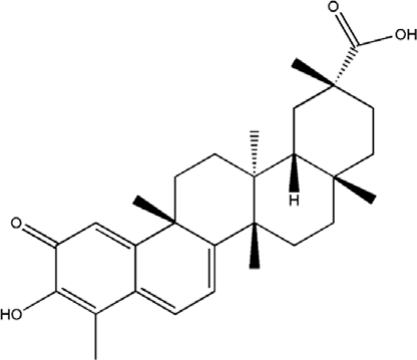	1. Inhibit bone metastasis of prostate cancer	Kuchta et al. (2017)
		2. Inhibit the VEGF pathway of bone marrow-derived endothelial progenitor cells	
Perillaldehyde	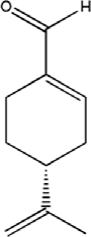	1. Inhibit bone metastasis of prostate cancer	Z. Lin et al. (2022)
		2. Inhibit the RANKL-induced osteoclastogenesis	
Aldehydic components of cinnamon bark extract	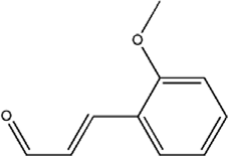	1. Inhibit bone metastasis of prostate cancer	Tsuji-Naito (2008)
		2. Inhibit the RANKL-induced osteoclastogenesis	

## 3 Conclusion

The concept of PMBN may be convenient for the illustration of the predilection of PCa bone metastasis. In brief, after stimulation by TDSF, MICs will migrate to the target niche and activate chemokines, which further induces the BMDC to MDSC in bone. Fibronectin, integrin, and VEGFR promote the recruitment and fusion of PMBN. As BMDC comes from the bone, the formation of PMBN is easier than any other PMN. After maturation of PMBN, MICs will activate in bone and induce tumor cell metastasis from primary prostate to bone, which affects the balance between osteoblast, and osteoclast thereby leading to bone metastasis. Moreover, TCM has potential regulation in PMBN. However, PMBN is just a hypothesis for now and further basic research is necessary to determine the cause of the predilection of PCa bone metastasis.

## Abbreviations

BMP, bone morphogenetic protein; BMDC, bone marrow-derived cells; CSCs, cancer stem cells; DTCs, disseminated tumor cells; ECM, extracellular matrix; EMT, epithelial–mesenchymal transition; HSPC, hematopoietic stem/progenitor cells; HSC, hematopoietic progenitor cells; MET, mesenchymal- epithelial transition; MDSC, Myeloid-derived suppressor cells; MICs, migration of Metastasis-initiating cells; PCa, Prostate cancer; PMN, pre-metastatic niche; PMBN: pre-metastatic bone niche; TME, tumor-micro-environment; TDSF, tumor-derived soluble factors; TDEs, tumor-derive exosomes; TAMs, tumor-associated macrophages; VEGF, vascular endothelial growth factor; VLA4, integrin alpha4beta1; VEGFR, vascular endothelial growth factor receptor.

## References

[B1] Aguilar E. Marin de Mas I. Zodda E. Marin S. Morrish F. Selivanov V. (2016). Metabolic reprogramming and dependencies associated with epithelial cancer stem cells independent of the epithelial-mesenchymal transition program. STEM CELLS 34 (5), 1163–1176. 10.1002/stem.2286 27146024 PMC4860823

[B2] Ahmadzadeh A. Kast R. E. Ketabchi N. Shahrabi S. Shahjahani M. Jaseb K. (2015). Regulatory effect of chemokines in bone marrow niche. Cell. Tissue Res. 361 (2), 401–410. 10.1007/s00441-015-2129-4 25715759

[B3] Allard M. Oger R. Vignard V. Percier J. M. Fregni G. Périer A. (2011). Serum soluble HLA-E in melanoma: A new potential immune-related marker in cancer. PLoS One 6 (6), e21118. 10.1371/journal.pone.0021118 21712991 PMC3119680

[B4] An G. Wu F. Huang S. Feng L. Bai J. Gu S. (2019). Effects of CCL5 on the biological behavior of breast cancer and the mechanisms of its interaction with tumor-associated macrophages. Oncol. Rep. 42 (6), 2499–2511. 10.3892/or.2019.7344 31578575 PMC6826325

[B5] Arbiser J. L. Petros J. Klafter R. Govindajaran B. McLaughlin E. R. Brown L. F. (2002). Reactive oxygen generated by Nox1 triggers the angiogenic switch. Proc. Natl. Acad. Sci. U. S. A. 99 (2), 715–720. 10.1073/pnas.022630199 11805326 PMC117371

[B6] Aufderklamm S. Hennenlotter J. Leidenberger P. Rausch S. Hohneder A. Kühs U. (2018). Systemic alterations of Wnt inhibitors in patients with prostate cancer and bone metastases. Dis. Markers 2018, 1874598. 10.1155/2018/1874598 30116403 PMC6079590

[B7] Aurrand-Lions M. Mancini S. J. C. (2018). Murine bone marrow niches from hematopoietic stem cells to B cells. Int. J. Mol. Sci. 19 (8), E2353. 10.3390/ijms19082353 30103411 PMC6121419

[B8] Banzola I. Mengus C. Wyler S. Hudolin T. Manzella G. Chiarugi A. (2018). Expression of indoleamine 2, 3-dioxygenase induced by IFN-γ and TNF-α as potential biomarker of prostate cancer progression. Front. Immunol. 9, 1051. 10.3389/fimmu.2018.01051 29896191 PMC5986916

[B9] Berruti A. Dogliotti L. Tucci M. Tarabuzzi R. Fontana D. Angeli A. (2001). Metabolic bone disease induced by prostate cancer: Rationale for the use of bisphosphonates. J. Urology 166 (6), 2023–2031. 10.1097/00005392-200112000-00004 11696699

[B10] Bizzarro V. Belvedere R. Migliaro V. Romano E. Parente L. Petrella A. (2017). Hypoxia regulates ANXA1 expression to support prostate cancer cell invasion and aggressiveness. Cell. adh. Migr. 11 (3), 247–260. 10.1080/19336918.2016.1259056 27834582 PMC5479448

[B11] Bronte V. Brandau S. Chen S. H. Colombo M. P. Frey A. B. Greten T. F. (2016). Recommendations for myeloid-derived suppressor cell nomenclature and characterization standards. Nat. Commun. 7, 12150. 10.1038/ncomms12150 27381735 PMC4935811

[B12] Cameron M. D. Schmidt E. E. Kerkvliet N. Nadkarni K. V. Morris V. L. Groom A. C. (2000). Temporal progression of metastasis in lung: Cell survival, dormancy, and location dependence of metastatic inefficiency. Cancer Res. 60 (9), 2541–2546. 10811137

[B13] Casimiro S. Ferreira A. R. Mansinho A. Alho I. Costa L. (2016). Molecular mechanisms of bone metastasis: Which targets came from the bench to the bedside? Int. J. Mol. Sci. 17 (9), E1415. 10.3390/ijms17091415 27618899 PMC5037694

[B14] Celià-Terrassa T. Kang Y. (2016). Distinctive properties of metastasis-initiating cells. Genes. Dev. 30 (8), 892–908. 10.1101/gad.277681.116 27083997 PMC4840296

[B15] Chalmin F. Ladoire S. Mignot G. Vincent J. Bruchard M. Remy-Martin J. P. (2010). Membrane-associated Hsp72 from tumor-derived exosomes mediates STAT3-dependent immunosuppressive function of mouse and human myeloid-derived suppressor cells. J. Clin. Invest. 120 (2), 457–471. 10.1172/jci40483 20093776 PMC2810085

[B16] Chang A. C. Chen P. C. Lin Y. F. Su C. M. Liu J. F. Lin T. H. (2018). Osteoblast-secreted WISP-1 promotes adherence of prostate cancer cells to bone via the VCAM-1/integrin α4β1 system. Cancer Lett. 426, 47–56. 10.1016/j.canlet.2018.03.050 29627497

[B17] Chen C. Yao X. Xu Y. Zhang Q. Wang H. Zhao L. (2019). Dahuang Zhechong Pill suppresses colorectal cancer liver metastasis via ameliorating exosomal CCL2 primed pre-metastatic niche. J. Ethnopharmacol. 238, 111878. 10.1016/j.jep.2019.111878 30986521

[B18] Chen E. I. Hewel J. Krueger J. S. Tiraby C. Weber M. R. Kralli A. (2007). Adaptation of energy metabolism in breast cancer brain metastases. Cancer Res. 67 (4), 1472–1486. 10.1158/0008-5472.Can-06-3137 17308085

[B19] Chen J. De S. Brainard J. Byzova T. V. (2004). Metastatic properties of prostate cancer cells are controlled by VEGF. Cell. Commun. Adhes. 11 (1), 1–11. 10.1080/15419060490471739 15500293

[B20] Chen P. C. Tang C. H. Lin L. W. Tsai C. H. Chu C. Y. Lin T. H. (2017a). Thrombospondin-2 promotes prostate cancer bone metastasis by the up-regulation of matrix metalloproteinase-2 through down-regulating miR-376c expression. J. Hematol. Oncol. 10 (1), 33. 10.1186/s13045-017-0390-6 28122633 PMC5264454

[B21] Chen X. W. Yu T. J. Zhang J. Li Y. Chen H. L. Yang G. F. (2017b). CYP4A in tumor-associated macrophages promotes pre-metastatic niche formation and metastasis. ONCOGENE 36 (35), 5045–5057. 10.1038/onc.2017.118 28481877 PMC5582214

[B22] Cheng X. Wang Z. (2021). Immune modulation of metastatic niche formation in the bone. Front. Immunol. 12, 765994. 10.3389/fimmu.2021.765994 34745140 PMC8564379

[B23] Chin A. R. Wang S. E. (2016). Cancer tills the premetastatic field: Mechanistic basis and clinical implications. Clin. Cancer Res. 22 (15), 3725–3733. 10.1158/1078-0432.Ccr-16-0028 27252414 PMC4970897

[B24] Chinni S. R. Sivalogan S. Dong Z. Filho J. C. Deng X. Bonfil R. D. (2006). CXCL12/CXCR4 signaling activates akt-1 and MMP-9 expression in prostate cancer cells: The role of bone microenvironment-associated CXCL12. PROSTATE 66 (1), 32–48. 10.1002/pros.20318 16114056

[B25] Coniglio S. J. (2018). Role of tumor-derived chemokines in osteolytic bone metastasis. Front. Endocrinol. 9, 313. 10.3389/fendo.2018.00313 PMC5999726 29930538

[B26] Conley-LaComb M. K. Saliganan A. Kandagatla P. Chen Y. Q. Cher M. L. Chinni S. R. (2013). PTEN loss mediated Akt activation promotes prostate tumor growth and metastasis via CXCL12/CXCR4 signaling. Mol. Cancer 12 (1), 85. 10.1186/1476-4598-12-85 23902739 PMC3751767

[B27] Cox T. R. Bird D. Baker A. M. Barker H. E. Ho M. W. Lang G. (2013). LOX-mediated collagen crosslinking is responsible for fibrosis-enhanced metastasis. Cancer Res. 73 (6), 1721–1732. 10.1158/0008-5472.Can-12-2233 23345161 PMC3672851

[B28] Crane C. A. Ahn B. J. Han S. J. Parsa A. T. (2012). Soluble factors secreted by glioblastoma cell lines facilitate recruitment, survival, and expansion of regulatory T cells: Implications for immunotherapy. Neuro. Oncol. 14 (5), 584–595. 10.1093/neuonc/nos014 22406925 PMC3337302

[B29] Dai J. Keller J. Zhang J. Lu Y. Yao Z. Keller E. T. (2005). Bone morphogenetic protein-6 promotes osteoblastic prostate cancer bone metastases through a dual mechanism. Cancer Res. 65 (18), 8274–8285. 10.1158/0008-5472.Can-05-1891 16166304

[B30] Dai Y. Ren D. Yang Q. Cui Y. Guo W. Lai Y. (2017). The TGF-β signalling negative regulator PICK1 represses prostate cancer metastasis to bone. Br. J. Cancer 117 (5), 685–694. 10.1038/bjc.2017.212 28697177 PMC5572169

[B31] Deep G. Jain A. Kumar A. Agarwal C. Kim S. Leevy W. M. (2020). Exosomes secreted by prostate cancer cells under hypoxia promote matrix metalloproteinases activity at pre-metastatic niches. Mol. Carcinog. 59 (3), 323–332. 10.1002/mc.23157 31943365 PMC7189745

[B32] Deepak P. Acharya A. (2010). Anti-tumor immunity and mechanism of immunosuppression mediated by tumor cells: Role of tumor-derived soluble factors and cytokines. Int. Rev. Immunol. 29 (4), 421–458. 10.3109/08830185.2010.483027 20635882

[B33] Ding H. Zhao L. Dai S. Li L. Wang F. Shan B. (2016). CCL5 secreted by tumor associated macrophages may be a new target in treatment of gastric cancer. Biomed. Pharmacother. 77, 142–149. 10.1016/j.biopha.2015.12.004 26796278

[B34] Du C. Wang Y. (2011). The immunoregulatory mechanisms of carcinoma for its survival and development. J. Exp. Clin. Cancer Res. 30 (1), 12. 10.1186/1756-9966-30-12 21255410 PMC3031251

[B35] Dutta A. Li J. Lu H. Akech J. Pratap J. Wang T. (2014). Integrin αvβ6 promotes an osteolytic program in cancer cells by upregulating MMP2. Cancer Res. 74 (5), 1598–1608. 10.1158/0008-5472.Can-13-1796 24385215 PMC3967411

[B36] Erler J. T. Bennewith K. L. Cox T. R. Lang G. Bird D. Koong A. (2009). Hypoxia-induced lysyl oxidase is a critical mediator of bone marrow cell recruitment to form the premetastatic niche. CANCER Cell. 15 (1), 35–44. 10.1016/j.ccr.2008.11.012 19111879 PMC3050620

[B37] Furesi G. Rauner M. Hofbauer L. C. (2021). Emerging players in prostate cancer-bone niche communication. Trends Cancer 7 (2), 112–121. 10.1016/j.trecan.2020.09.006 33274720

[B38] Gabrilovich D. I. (2017). Myeloid-derived suppressor cells. Cancer Immunol. Res. 5 (1), 3–8. 10.1158/2326-6066.Cir-16-0297 28052991 PMC5426480

[B39] Gao H. Chakraborty G. Lee-Lim A. P. Mo Q. Decker M. Vonica A. (2012). The BMP inhibitor Coco reactivates breast cancer cells at lung metastatic sites. Cell. 150 (4), 764–779. 10.1016/j.cell.2012.06.035 22901808 PMC3711709

[B40] Gartland A. Erler J. T. Cox T. R. (2016). The role of lysyl oxidase, the extracellular matrix and the pre-metastatic niche in bone metastasis. J. Bone Oncol. 5 (3), 100–103. 10.1016/j.jbo.2016.04.001 27761366 PMC5063254

[B41] Ghajar C. M. Peinado H. Mori H. Matei I. R. Evason K. J. Brazier H. (2013). The perivascular niche regulates breast tumour dormancy. Nat. Cell. Biol. 15 (7), 807–817. 10.1038/ncb2767 23728425 PMC3826912

[B42] Giancotti F. G. (2013). Mechanisms governing metastatic dormancy and reactivation. Cell. 155 (4), 750–764. 10.1016/j.cell.2013.10.029 24209616 PMC4354734

[B43] Giles A. J. Reid C. M. Evans J. D. Murgai M. Vicioso Y. Highfill S. L. (2016). Activation of hematopoietic stem/progenitor cells promotes immunosuppression within the pre-metastatic niche. Cancer Res. 76 (6), 1335–1347. 10.1158/0008-5472.Can-15-0204 26719537 PMC4794356

[B44] Graham T. R. Agrawal K. C. Abdel-Mageed A. B. (2010). Independent and cooperative roles of tumor necrosis factor-alpha, nuclear factor-kappaB, and bone morphogenetic protein-2 in regulation of metastasis and osteomimicry of prostate cancer cells and differentiation and mineralization of MC3T3-E1 osteoblast-like cells. Cancer Sci. 101 (1), 103–111. 10.1111/j.1349-7006.2009.01356.x 19811499 PMC3092127

[B45] Hall C. L. Daignault S. D. Shah R. B. Pienta K. J. Keller E. T. (2008). Dickkopf-1 expression increases early in prostate cancer development and decreases during progression from primary tumor to metastasis. PROSTATE 68 (13), 1396–1404. 10.1002/pros.20805 18561248 PMC3260942

[B46] Han L. Mei J. Ma J. Wang F. Gu Z. Li J. (2020). Cinnamaldehyde induces endogenous apoptosis of the prostate cancer-associated fibroblasts via interfering the Glutathione-associated mitochondria function. Med. Oncol. 37 (10), 91. 10.1007/s12032-020-01417-2 32960365

[B47] Hiraga T. (2019). Bone metastasis: Interaction between cancer cells and bone microenvironment. J. Oral Biosci. 61 (2), 95–98. 10.1016/j.job.2019.02.002 31109867

[B48] Hiraga T. Myoui A. Hashimoto N. Sasaki A. Hata K. Morita Y. (2012). Bone-derived IGF mediates crosstalk between bone and breast cancer cells in bony metastases. Cancer Res. 72 (16), 4238–4249. 10.1158/0008-5472.Can-11-3061 22738911 PMC3438359

[B49] Hoshino A. Costa-Silva B. Shen T. L. Rodrigues G. Hashimoto A. Tesic Mark M. (2015). Tumour exosome integrins determine organotropic metastasis. NATURE 527 (7578), 329–335. 10.1038/nature15756 26524530 PMC4788391

[B50] Huang R. Wang S. Wang N. Zheng Y. Zhou J. Yang B. (2020). CCL5 derived from tumor-associated macrophages promotes prostate cancer stem cells and metastasis via activating β-catenin/STAT3 signaling. Cell. Death Dis. 11 (4), 234. 10.1038/s41419-020-2435-y 32300100 PMC7162982

[B51] Huang S. Wa Q. Pan J. Peng X. Ren D. Huang Y. (2017). Downregulation of miR-141-3p promotes bone metastasis via activating NF-κB signaling in prostate cancer. J. Exp. Clin. Cancer Res. 36 (1), 173. 10.1186/s13046-017-0645-7 29202848 PMC5716366

[B52] Huang S. Wa Q. Pan J. Peng X. Ren D. Li Q. (2018). Transcriptional downregulation of miR-133b by REST promotes prostate cancer metastasis to bone via activating TGF-β signaling. Cell. Death Dis. 9 (7), 779. 10.1038/s41419-018-0807-3 30006541 PMC6045651

[B53] Ishii H. Vodnala S. K. Achyut B. R. So J. Y. Hollander M. C. Greten T. F. (2018). miR-130a and miR-145 reprogram Gr-1(+)CD11b(+) myeloid cells and inhibit tumor metastasis through improved host immunity. Nat. Commun. 9 (1), 2611. 10.1038/s41467-018-05023-9 29973593 PMC6031699

[B54] Jin T. Xu X. Hereld D. (2008). Chemotaxis, chemokine receptors and human disease. CYTOKINE 44 (1), 1–8. 10.1016/j.cyto.2008.06.017 18722135 PMC2613022

[B55] Kai F. Drain A. P. Weaver V. M. (2019). The extracellular matrix modulates the metastatic journey. Dev. Cell. 49 (3), 332–346. 10.1016/j.devcel.2019.03.026 31063753 PMC6527347

[B56] Kaplan R. N. Psaila B. Lyden D. (2006a). Bone marrow cells in the 'pre-metastatic niche': Within bone and beyond. Cancer Metastasis Rev. 25 (4), 521–529. 10.1007/s10555-006-9036-9 17186383

[B57] Kaplan R. N. Rafii S. Lyden D. (2006b). Preparing the "soil": The premetastatic niche. Cancer Res. 66 (23), 11089–11093. 10.1158/0008-5472.Can-06-2407 17145848 PMC2952469

[B58] Kaplan R. N. Riba R. D. Zacharoulis S. Bramley A. H. Vincent L. Costa C. (2005). VEGFR1-positive haematopoietic bone marrow progenitors initiate the pre-metastatic niche. Nature 438 (7069), 820–827. 10.1038/nature04186 16341007 PMC2945882

[B59] King H. W. Michael M. Z. Gleadle J. M. (2012). Hypoxic enhancement of exosome release by breast cancer cells. BMC CANCER 12, 421. 10.1186/1471-2407-12-421 22998595 PMC3488584

[B60] Kitagawa Y. Dai J. Zhang J. Keller J. M. Nor J. Yao Z. (2005). Vascular endothelial growth factor contributes to prostate cancer-mediated osteoblastic activity. Cancer Res. 65 (23), 10921–10929. 10.1158/0008-5472.Can-05-1809 16322239

[B61] Koh B. I. Kang Y. (2012). The pro-metastatic role of bone marrow-derived cells: A focus on MSCs and regulatory T cells. EMBO Rep. 13 (5), 412–422. 10.1038/embor.2012.41 22473297 PMC3343352

[B62] Kuchta K. Xiang Y. Huang S. Tang Y. Peng X. Wang X. (2017). Celastrol, an active constituent of the TCM plant Tripterygium wilfordii Hook.f., inhibits prostate cancer bone metastasis. Prostate Cancer Prostatic Dis. 20 (2), 250. 10.1038/pcan.2017.11 28349980

[B63] Kucia M. Reca R. Miekus K. Wanzeck J. Wojakowski W. Janowska-Wieczorek A. (2005). Trafficking of normal stem cells and metastasis of cancer stem cells involve similar mechanisms: Pivotal role of the SDF-1-CXCR4 axis. STEM CELLS 23 (7), 879–894. 10.1634/stemcells.2004-0342 15888687

[B64] Lang C. Dai Y. Wu Z. Yang Q. He S. Zhang X. (2020). SMAD3/SP1 complex-mediated constitutive active loop between lncRNA PCAT7 and TGF-β signaling promotes prostate cancer bone metastasis. Mol. Oncol. 14 (4), 808–828. 10.1002/1878-0261.12634 31925912 PMC7138406

[B65] Lawson D. A. Bhakta N. R. Kessenbrock K. Prummel K. D. Yu Y. Takai K. (2015). Single-cell analysis reveals a stem-cell program in human metastatic breast cancer cells. NATURE 526 (7571), 131–135. 10.1038/nature15260 26416748 PMC4648562

[B66] Li K. Shi H. Zhang B. Ou X. Ma Q. Chen Y. (2021). Myeloid-derived suppressor cells as immunosuppressive regulators and therapeutic targets in cancer. Signal Transduct. Target. Ther. 6 (1), 362. 10.1038/s41392-021-00670-9 34620838 PMC8497485

[B67] Lin S. Sun L. Lyu X. Ai X. Du D. Su N. (2017). Lactate-activated macrophages induced aerobic glycolysis and epithelial-mesenchymal transition in breast cancer by regulation of CCL5-CCR5 axis: A positive metabolic feedback loop. Oncotarget 8 (66), 110426–110443. 10.18632/oncotarget.22786 29299159 PMC5746394

[B68] Lin Z. Huang S. LingHu X. Wang Y. Wang B. Zhong S. (2022). Perillaldehyde inhibits bone metastasis and receptor activator of nuclear factor-κB ligand (RANKL) signaling-induced osteoclastogenesis in prostate cancer cell lines. Bioengineered 13 (2), 2710–2719. 10.1080/21655979.2021.2001237 34738877 PMC8973720

[B69] Liu X. Chen L. Fan Y. Hong Y. Yang X. Li Y. (2019). IFITM3 promotes bone metastasis of prostate cancer cells by mediating activation of the TGF-β signaling pathway. Cell. Death Dis. 10 (7), 517. 10.1038/s41419-019-1750-7 31273201 PMC6609682

[B70] Liu Y. Cao X. (2016). Organotropic metastasis: Role of tumor exosomes. Cell. Res. 26 (2), 149–150. 10.1038/cr.2015.153 26704450 PMC4746605

[B71] Lu X. Mu E. Wei Y. Riethdorf S. Yang Q. Yuan M. (2011). VCAM-1 promotes osteolytic expansion of indolent bone micrometastasis of breast cancer by engaging α4β1-positive osteoclast progenitors. CANCER Cell. 20 (6), 701–714. 10.1016/j.ccr.2011.11.002 22137794 PMC3241854

[B72] Luzzi K. J. MacDonald I. C. Schmidt E. E. Kerkvliet N. Morris V. L. Chambers A. F. (1998). Multistep nature of metastatic inefficiency: Dormancy of solitary cells after successful extravasation and limited survival of early micrometastases. Am. J. Pathol. 153 (3), 865–873. 10.1016/s0002-9440(10)65628-3 9736035 PMC1853000

[B73] Lynch M. E. Chiou A. E. Lee M. J. Marcott S. C. Polamraju P. V. Lee Y. (2016). Three-dimensional mechanical loading modulates the osteogenic response of mesenchymal stem cells to tumor-derived soluble signals. Tissue Eng. Part A 22 (15-16), 1006–1015. 10.1089/ten.TEA.2016.0153 27401765 PMC4991606

[B74] Malanchi I. Santamaria-Martínez A. Susanto E. Peng H. Lehr H. A. Delaloye J. F. (2011). Interactions between cancer stem cells and their niche govern metastatic colonization. NATURE 481 (7379), 85–89. 10.1038/nature10694 22158103

[B75] Martínez J. Silva S. Santibáñez J. F. (1996). Prostate-derived soluble factors block osteoblast differentiation in culture. J. Cell. Biochem. 61 (1), 18–25. 10.1002/(sici)1097-4644(19960401)61:1<18::aid-jcb3>3.0.co;2-5 8726351

[B76] Maxwell P. J. Neisen J. Messenger J. Waugh D. J. (2014). Tumor-derived CXCL8 signaling augments stroma-derived CCL2-promoted proliferation and CXCL12-mediated invasion of PTEN-deficient prostate cancer cells. Oncotarget 5 (13), 4895–4908. 10.18632/oncotarget.2052 24970800 PMC4148108

[B77] Mazzone E. Preisser F. Nazzani S. Tian Z. Bandini M. Gandaglia G. (2018). Location of metastases in contemporary prostate cancer patients affects cancer-specific mortality. Clin. Genitourin. Cancer 16 (5), 376–384. e371. 10.1016/j.clgc.2018.05.016 29954690

[B78] Meng W. Xue S. Chen Y. (2018). The role of CXCL12 in tumor microenvironment. GENE 641, 105–110. 10.1016/j.gene.2017.10.015 29017963

[B79] Morales J. K. Kmieciak M. Knutson K. L. Bear H. D. Manjili M. H. (2010). GM-CSF is one of the main breast tumor-derived soluble factors involved in the differentiation of CD11b-Gr1- bone marrow progenitor cells into myeloid-derived suppressor cells. Breast Cancer Res. Treat. 123 (1), 39–49. 10.1007/s10549-009-0622-8 PMC3095485 19898981

[B80] Morrissey C. True L. D. Roudier M. P. Coleman I. M. Hawley S. Nelson P. S. (2008). Differential expression of angiogenesis associated genes in prostate cancer bone, liver and lymph node metastases. Clin. Exp. Metastasis 25 (4), 377–388. 10.1007/s10585-007-9116-4 17972146

[B81] Mouw J. K. Ou G. Weaver V. M. (2014). Extracellular matrix assembly: A multiscale deconstruction. Nat. Rev. Mol. Cell. Biol. 15 (12), 771–785. 10.1038/nrm3902 25370693 PMC4682873

[B82] Mukaida N. Zhang D. Sasaki S. I. (2020). Emergence of cancer-associated fibroblasts as an indispensable cellular player in bone metastasis process. Cancers (Basel) 12 (10), E2896. 10.3390/cancers12102896 33050237 PMC7600711

[B83] Mundy G. R. (2002). Metastasis to bone: Causes, consequences and therapeutic opportunities. Nat. Rev. Cancer 2 (8), 584–593. 10.1038/nrc867 12154351

[B84] Murgai M. Ju W. Eason M. Kline J. Beury D. W. Kaczanowska S. (2017). KLF4-dependent perivascular cell plasticity mediates pre-metastatic niche formation and metastasis. Nat. Med. 23 (10), 1176–1190. 10.1038/nm.4400 28920957 PMC5724390

[B85] Nguyen D. P. Li J. Tewari A. K. (2014). Inflammation and prostate cancer: The role of interleukin 6 (IL-6). BJU Int. 113 (6), 986–992. 10.1111/bju.12452 24053309

[B86] Packard B. Z. Lee S. S. Remold-O'Donnell E. Komoriya A. (1995). A serpin from human tumor cells with direct lymphoid immunomodulatory activity: Mitogenic stimulation of human tumor-infiltrating lymphocytes. Biochim. Biophys. Acta 1269 (1), 41–50. 10.1016/0167-4889(95)00113-7 7578269

[B87] Paget S. (1989). The distribution of secondary growths in cancer of the breast. 1889. Cancer Metastasis Rev. 8 (2), 98–101. 2673568

[B88] Panigrahi G. K. Praharaj P. P. Peak T. C. Long J. Singh R. Rhim J. S. (2018). Hypoxia-induced exosome secretion promotes survival of African-American and Caucasian prostate cancer cells. Sci. Rep. 8 (1), 3853. 10.1038/s41598-018-22068-4 29497081 PMC5832762

[B89] Peinado H. Zhang H. Matei I. R. Costa-Silva B. Hoshino A. Rodrigues G. (2017). Pre-metastatic niches: Organ-specific homes for metastases. Nat. Rev. Cancer 17 (5), 302–317. 10.1038/nrc.2017.6 28303905

[B90] Pernar C. H. Ebot E. M. Wilson K. M. Mucci L. A. (2018). The epidemiology of prostate cancer. Cold Spring Harb. Perspect. Med. 8 (12), a030361. 10.1101/cshperspect.a030361 29311132 PMC6280714

[B91] Portal-Núñez S. Mediero A. Esbrit P. Sánchez-Pernaute O. Largo R. Herrero-Beaumont G. (2017). Unexpected bone formation produced by RANKL blockade. Trends Endocrinol. Metab. 28 (10), 695–704. 10.1016/j.tem.2017.06.003 28733136

[B92] Psaila B. Kaplan R. N. Port E. R. Lyden D. (2006). Priming the 'soil' for breast cancer metastasis: The pre-metastatic niche. Breast Dis. 26, 65–74. 10.3233/bd-2007-26106 17473366

[B93] Ren D. Yang Q. Dai Y. Guo W. Du H. Song L. (2017). Oncogenic miR-210-3p promotes prostate cancer cell EMT and bone metastasis via NF-κB signaling pathway. Mol. Cancer 16 (1), 117. 10.1186/s12943-017-0688-6 28693582 PMC5504657

[B94] Riihimäki M. Thomsen H. Sundquist K. Sundquist J. Hemminki K. (2018). Clinical landscape of cancer metastases. Cancer Med. 7 (11), 5534–5542. 10.1002/cam4.1697 30328287 PMC6246954

[B95] Rosanò L. Spinella F. Bagnato A. (2013). Endothelin 1 in cancer: Biological implications and therapeutic opportunities. Nat. Rev. Cancer 13 (9), 637–651. 10.1038/nrc3546 23884378

[B96] Sasaki S. Baba T. Nishimura T. Hayakawa Y. Hashimoto S. Gotoh N. (2016). Essential roles of the interaction between cancer cell-derived chemokine, CCL4, and intra-bone CCR5-expressing fibroblasts in breast cancer bone metastasis. Cancer Lett. 378 (1), 23–32. 10.1016/j.canlet.2016.05.005 27177471

[B97] Schwaninger R. Rentsch C. A. Wetterwald A. van der Horst G. van Bezooijen R. L. van der Pluijm G. (2007). Lack of noggin expression by cancer cells is a determinant of the osteoblast response in bone metastases. Am. J. Pathol. 170 (1), 160–175. 10.2353/ajpath.2007.051276 17200191 PMC1762703

[B98] Shen X. Falzon M. (2003). Parathyroid hormone-related protein upregulates integrin expression via an intracrine pathway in PC-3 prostate cancer cells. Regul. Pept. 113 (1-3), 17–29. 10.1016/s0167-0115(02)00293-8 12686457

[B99] Shibue T. Weinberg R. A. (2009). Integrin beta1-focal adhesion kinase signaling directs the proliferation of metastatic cancer cells disseminated in the lungs. Proc. Natl. Acad. Sci. U. S. A. 106 (25), 10290–10295. 10.1073/pnas.0904227106 19502425 PMC2700942

[B100] Shimabukuro-Vornhagen A. Draube A. Liebig T. M. Rothe A. Kochanek M. von Bergwelt-Baildon M. S. (2012). The immunosuppressive factors IL-10, TGF-β, and VEGF do not affect the antigen-presenting function of CD40-activated B cells. J. Exp. Clin. Cancer Res. 31 (1), 47. 10.1186/1756-9966-31-47 22592077 PMC3443023

[B101] Sosa M. S. Bragado P. Aguirre-Ghiso J. A. (2014). Mechanisms of disseminated cancer cell dormancy: An awakening field. Nat. Rev. Cancer 14 (9), 611–622. 10.1038/nrc3793 25118602 PMC4230700

[B102] Sun Y. X. Schneider A. Jung Y. Wang J. Dai J. Wang J. (2005). Skeletal localization and neutralization of the SDF-1(CXCL12)/CXCR4 axis blocks prostate cancer metastasis and growth in osseous sites *in vivo* . J. Bone Min. Res. 20 (2), 318–329. 10.1359/jbmr.041109 15647826

[B103] Sun Y. X. Wang J. Shelburne C. E. Lopatin D. E. Chinnaiyan A. M. Rubin M. A. (2003). Expression of CXCR4 and CXCL12 (SDF-1) in human prostate cancers (PCa) *in vivo* . J. Cell. Biochem. 89 (3), 462–473. 10.1002/jcb.10522 12761880

[B104] Tang K. D. Holzapfel B. M. Liu J. Lee T. K. Ma S. Jovanovic L. (2016). Tie-2 regulates the stemness and metastatic properties of prostate cancer cells. Oncotarget 7 (3), 2572–2584. 10.18632/oncotarget.3950 25978029 PMC4823056

[B105] Tang Y. Pan J. Huang S. Peng X. Zou X. Luo Y. (2018). Downregulation of miR-133a-3p promotes prostate cancer bone metastasis via activating PI3K/AKT signaling. J. Exp. Clin. Cancer Res. 37 (1), 160. 10.1186/s13046-018-0813-4 30021600 PMC6052526

[B106] Tang Y. Wu B. Huang S. Peng X. Li X. Huang X. (2019). Downregulation of miR-505-3p predicts poor bone metastasis-free survival in prostate cancer. Oncol. Rep. 41 (1), 57–66. 10.3892/or.2018.6826 30365141 PMC6278553

[B107] Thalmann G. N. Sikes R. A. Devoll R. E. Kiefer J. A. Markwalder R. Klima I. (1999). Osteopontin: Possible role in prostate cancer progression. Clin. Cancer Res. 5 (8), 2271–2277. 10473115

[B108] Tian S. Song X. Wang Y. Wang X. Mou Y. Chen Q. (2020). Chinese herbal medicine Baoyuan Jiedu decoction inhibits the accumulation of myeloid derived suppressor cells in pre-metastatic niche of lung via TGF-β/CCL9 pathway. Biomed. Pharmacother. 129, 110380. 10.1016/j.biopha.2020.110380 32554250

[B109] Toth R. K. Tran J. D. Muldong M. T. Nollet E. A. Schulz V. V. Jensen C. C. (2019). Hypoxia-induced PIM kinase and laminin-activated integrin α6 mediate resistance to PI3K inhibitors in bone-metastatic CRPC. Am. J. Clin. Exp. Urol. 7 (4), 297–312. 31511835 PMC6734039

[B110] Tsai J. H. Donaher J. L. Murphy D. A. Chau S. Yang J. (2012). Spatiotemporal regulation of epithelial-mesenchymal transition is essential for squamous cell carcinoma metastasis. CANCER Cell. 22 (6), 725–736. 10.1016/j.ccr.2012.09.022 23201165 PMC3522773

[B111] Tsuji-Naito K. (2008). Aldehydic components of cinnamon bark extract suppresses RANKL-induced osteoclastogenesis through NFATc1 downregulation. Bioorg. Med. Chem. 16 (20), 9176–9183. 10.1016/j.bmc.2008.09.036 18823786

[B112] Ugel S. De Sanctis F. Mandruzzato S. Bronte V. (2015). Tumor-induced myeloid deviation: When myeloid-derived suppressor cells meet tumor-associated macrophages. J. Clin. Invest. 125 (9), 3365–3376. 10.1172/jci80006 26325033 PMC4588310

[B113] Wa Q. Huang S. Pan J. Tang Y. He S. Fu X. (2019). miR-204-5p represses bone metastasis via inactivating NF-κB signaling in prostate cancer. Mol. Ther. Nucleic Acids 18, 567–579. 10.1016/j.omtn.2019.09.008 31678733 PMC6838892

[B114] Wa Q. Li L. Lin H. Peng X. Ren D. Huang Y. (2018). Downregulation of miR-19a-3p promotes invasion, migration and bone metastasis via activating TGF-β signaling in prostate cancer. Oncol. Rep. 39 (1), 81–90. 10.3892/or.2017.6096 29138858 PMC5783607

[B115] Wang N. Liu W. Zheng Y. Wang S. Yang B. Li M. (2018). CXCL1 derived from tumor-associated macrophages promotes breast cancer metastasis via activating NF-κB/SOX4 signaling. Cell. Death Dis. 9 (9), 880. 10.1038/s41419-018-0876-3 30158589 PMC6115425

[B116] Wang S. Wang N. Huang X. Yang B. Zheng Y. Zhang J. (2020). Baohuoside i suppresses breast cancer metastasis by downregulating the tumor-associated macrophages/C-X-C motif chemokine ligand 1 pathway. PHYTOMEDICINE 78, 153331. 10.1016/j.phymed.2020.153331 32911383

[B117] Wang W. Weng Y. Ren W. Zhang Z. Wang T. Wang J. (2015). Biological roles of human bone morphogenetic protein 9 in the bone microenvironment of human breast cancer MDA-MB-231 cells. Am. J. Transl. Res. 7 (9), 1660–1674. 26550465 PMC4626427

[B118] Wang Y. Ding Y. Guo N. Wang S. (2019). MDSCs: Key criminals of tumor pre-metastatic niche formation. Front. Immunol. 10, 172. 10.3389/fimmu.2019.00172 30792719 PMC6374299

[B119] Wen J. Huang G. Liu S. Wan J. Wang X. Zhu Y. (2020). Polymorphonuclear MDSCs are enriched in the stroma and expanded in metastases of prostate cancer. J. Pathol. Clin. Res. 6 (3), 171–177. 10.1002/cjp2.160 32149481 PMC7339199

[B120] Wortzel I. Dror S. Kenific C. M. Lyden D. (2019). Exosome-mediated metastasis: Communication from a distance. Dev. Cell. 49 (3), 347–360. 10.1016/j.devcel.2019.04.011 31063754

[B121] Wu S. Zheng Q. Xing X. Dong Y. Wang Y. You Y. (2018). Matrix stiffness-upregulated LOXL2 promotes fibronectin production, MMP9 and CXCL12 expression and BMDCs recruitment to assist pre-metastatic niche formation. J. Exp. Clin. Cancer Res. 37 (1), 99. 10.1186/s13046-018-0761-z 29728125 PMC5935912

[B122] Xie F. Ling L. van Dam H. Zhou F. Zhang L. (2018). TGF-β signaling in cancer metastasis. Acta Biochim. Biophys. Sin. 50 (1), 121–132. 10.1093/abbs/gmx123 29190313

[B123] Ye Y. Li S. L. Ma Y. Y. Diao Y. J. Yang L. Su M. Q. (2017). Exosomal miR-141-3p regulates osteoblast activity to promote the osteoblastic metastasis of prostate cancer. Oncotarget 8 (55), 94834–94849. 10.18632/oncotarget.22014 29212270 PMC5706916

[B124] Yoneda T. Hashimoto N. Hiraga T. (2003). Bisphosphonate actions on cancer. Calcif. Tissue Int. 73 (4), 315–318. 10.1007/s00223-002-0025-x 12874704

[B125] Yoneda T. (2011). Mechanism and strategy for treatment of cancer metastasis to bone. Gan Kagaku Ryoho. 38 (6), 877–884. 21677475

[B126] Zang L. Ma M. Hu J. Qiu H. Huang B. Chu T. (2015). The effects of lung and prostate cancer bone metastasis on serum osteoprotegerin levels: A meta-analysis. Sci. Rep. 5, 18324. 10.1038/srep18324 26671549 PMC4680868

[B127] Zhang C. Zhou C. Wu X. J. Yang M. Yang Z. H. Xiong H. Z. (2014). Human CD133-positive hematopoietic progenitor cells initiate growth and metastasis of colorectal cancer cells. CARCINOGENESIS 35 (12), 2771–2777. 10.1093/carcin/bgu192 25269803

[B128] Zhang J. Han X. Shi H. Gao Y. Qiao X. Li H. (2020). Lung resided monocytic myeloid-derived suppressor cells contribute to premetastatic niche formation by enhancing MMP-9 expression. Mol. Cell. Probes 50, 101498. 10.1016/j.mcp.2019.101498 31891749

[B129] Zhang J. J. Zhou X. H. Zhou Y. Wang Y. G. Qian B. Z. He A. N. (2019). Bufalin suppresses the migration and invasion of prostate cancer cells through HOTAIR, the sponge of miR-520b. Acta Pharmacol. Sin. 40 (9), 1228–1236. 10.1038/s41401-019-0234-8 31028291 PMC6786369

[B130] Zhang S. Zhong M. Wang C. Xu Y. Gao W. Q. Zhang Y. (2018). CCL5-deficiency enhances intratumoral infiltration of CD8(+) T cells in colorectal cancer. Cell. Death Dis. 9 (7), 766. 10.1038/s41419-018-0796-2 29991744 PMC6039518

[B131] Zhao Z. Li E. Luo L. Zhao S. Liu L. Wang J. (2020). A PSCA/PGRN-NF-κB-Integrin-α4 Axis promotes prostate cancer cell adhesion to bone marrow endothelium and enhances metastatic potential. Mol. Cancer Res. 18 (3), 501–513. 10.1158/1541-7786.Mcr-19-0278 31722969

[B132] Zheng Y. Wang N. Wang S. Yang B. Situ H. Zhong L. (2020). XIAOPI formula inhibits the pre-metastatic niche formation in breast cancer via suppressing TAMs/CXCL1 signaling. Cell. Commun. Signal. 18 (1), 48. 10.1186/s12964-020-0520-6 32213179 PMC7098160

[B133] Zhu X. Zhou Y. Xu Q. Wu J. (2017). Traditional Chinese medicine Jianpi Bushen therapy suppresses the onset of pre-metastatic niche in a murine model of spontaneous lung metastasis. Biomed. Pharmacother. 86, 434–440. 10.1016/j.biopha.2016.12.013 28012398

[B134] Zhuyan J. Chen M. Zhu T. Bao X. Zhen T. Xing K. (2020). Bone niche , Critical steps to tumor metastasis: alterations of tumor microenvironment and extracellular matrix in the formation of pre-metastatic and metastatic niche. Cell. Biosci. 10, 89. 10.1186/s13578-020-00453-9 32742634 PMC7388444

